# Cardiomiopatia Atrial Detectada por Eletrocardiograma: Associação com Acidente Vascular Encefálico em uma Coorte Eletrônica Brasileira

**DOI:** 10.36660/abc.20250625

**Published:** 2026-04-01

**Authors:** Filipe Antunes de Lêu, Gabriela Miana de Mattos Paixão, Ana Cecilia Oliveira, Carla Paula Moreira Soares, Paulo R. Gomes, Peter Macfarlane, Antônio Luiz Pinho Ribeiro

**Affiliations:** 1 Universidade Federal de Minas Gerais Belo Horizonte MG Brasil Universidade Federal de Minas Gerais, Belo Horizonte, MG – Brasil; 2 University of Glasgow School of Health and Wellbeing Glasgow Inglaterra University of Glasgow School of Health and Wellbeing, Glasgow,– Inglaterra

**Keywords:** Cardiomiopatias, Eletrocardiografia, Acidente Vascular Encefálico, Mortalidade, Estudos de Coortes

## Abstract

**Fundamento:**

A cardiomiopatia atrial (CA) é detectável pelo eletrocardiograma (ECG), e está relacionada à fisiopatologia do acidente vascular encefálico (AVE), independentemente de fibrilação atrial (FA).

**Objetivos:**

Avaliar a associação entre marcadores de ECG para CA (duração da onda P > 120 ms e a Força Terminal da Onda P em V1 > 4.000 μv × ms, PTFV1) e mortalidade e hospitalização por AVE.

**Métodos:**

Este estudo de coorte retrospectivo incluiu pacientes que realizaram ECGs entre 2006 e 2018. Foram critérios de inclusão: idade ≥ 40 anos, ritmo sinusal e ausência de histórico de AVE prévio ao ECG basal. Dados clínicos e de ECG foram pareados aos Sistemas de Informação de Mortalidade (SIM) e o Sistema de Informações Hospitalares (SIH). A regressão de Cox foi utilizada para calcular razões de risco (HRs), ajustadas para idade, sexo, fatores de risco cardiovascular e hipertrofia ventricular esquerda. A significância estatística foi estabelecida em p<0,05.

**Resultados:**

Dos 245588 pacientes, 26,3% apresentaram duração da onda P>120 ms, enquanto 10,1% apresentaram PTFV1 >4.000 µV·ms. O tempo médio de seguimento foi de 3,5 anos. A CA se associou à mortalidade e internação por AVE (HR 1,24; IC 95%, 1,12–1,36 para onda P >120 ms; p<0,001; HR 1,20; IC 95%, 1,05–1,38 para PTFV1 >4.000 µV·ms; p<0,001).

**Conclusão:**

Marcadores eletrocardiográficos de CA estão associados a morte ou internação por AVE, bem como a mortalidade cardiovascular e a incidência de FA em uma grande coorte brasileira, o que destaca seu potencial como marcadores prognósticos.

## Graphical abstract

**Figura Central:**
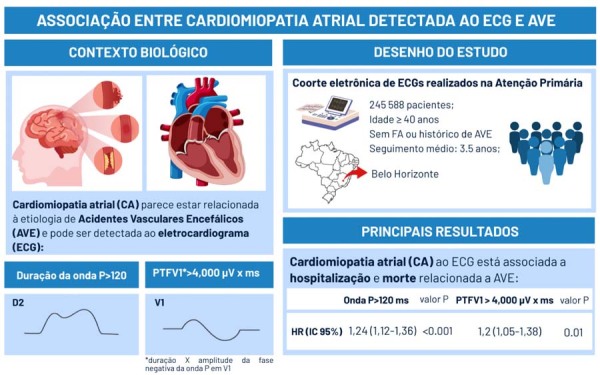
Cardiomiopatia Atrial Detectada por Eletrocardiograma: Associação com Acidente Vascular Encefálico em uma Coorte Eletrônica Brasileira

**Figura Central:** Cardiomiopatia Atrial Detectada por Eletrocardiograma: Associação com Acidente Vascular Encefálico em uma Coorte Eletrônica Brasileira

Figura elaborada pelos autores.

## Introdução

O acidente vascular encefálico (AVE) é a terceira maior causa de morte no mundo com impacto socioeconômico.^1 , 2^ O AVE isquêmico corresponde a 87% dos casos e sua etiologia está ligada a fenômenos aterotrombóticos e cardioembólicos.^3^ No entanto, cerca de 25% dos casos são classificados como criptogênicos, o que culmina em uma taxa de recorrência média de 3 a 6% de novo evento por ano.^4 , 5^ Acredita-se que parte dos casos de AVE criptogênico ocorre por mecanismos cardioembólicos ou ateroembólicos subjacentes.^6^ No contexto cardioembólico, a cardiomiopatia atrial (CA) pode ter associação causal relevante.^7^

A CA compreende um conjunto de alterações estruturais e funcionais do endomiocárdio atrial, que decorre de estímulos genéticos e ambientais^8 , 9^ e é considerada um precursor da fibrilação atrial (FA). No entanto, se sabe que a CA pode estar relacionada à ocorrência de eventos isquêmicos, independentemente da presença de FA.^7^ A CA leva à ruptura da homeostase, na qual são desencadeadas vias de sinalização fibroproliferativas que conduzem a diversas alterações a nível do cardiomiócito, com consequente disfunção endotelial, inflamação, hipocontratilidade e liberação de fatores pró-coagulantes, culminando no fenômeno da trombogênese.^8^ A FA potencializaria essa cascata à medida que acelera o remodelamento atrial e aumenta a estase sanguínea, mas não seria indispensável para a ocorrência do evento final.

O eletrocardiograma (ECG), exame simples e amplamente disponível, pode ser uma ferramenta útil para a detecção da CA , por meio da análise do aumento da duração da onda P e do aumento da Força Terminal da Onda P em V1 (PTFV1) - um produto entre duração e amplitude da fase negativa da onda P na derivação V1.^10 , 11^ Diversos estudos observacionais em bases estruturadas^12 - 14^ sugeriram uma relação entre a CA no ECG e desfechos adversos, como AVE isquêmico. Dessa forma, o objetivo deste estudo é avaliar a associação dos marcadores eletrocardiográficos de CA com a ocorrência de eventos cardiovasculares e morte em uma coorte eletrônica brasileira ( Figura Central ).

## Métodos

### Delineamento do estudo

Este estudo de coorte retrospectivo compreende a análise de um subgrupo da coorte *Clinical Outcomes in Digital Electrocardiography* (CODE), descrita previamente.^15^ Os achados foram reportados de acordo com as diretrizes STROBE ( *Strengthening the Reporting of Observational Studies in Epidemiology* ).^16^ Para a análise atual, pacientes que haviam sido submetidos a ECGs entre 2006 e 2018 na cidade de Belo Horizonte, Minas Gerais, Brasil, que é parte da Rede de Telessaúde de Minas Gerais (RTMG),^17^ participaram do estudo. Esse subgrupo de ECGs realizados em Belo Horizonte foi chamado de CODE-BH.

Os ECGs foram realizados pelo profissional da atenção primária, solicitados a critério do médico assistente, utilizando eletrocardiógrafos digitais da Tecnologia Eletrônica Brasileira, modelo ECGPC (São Paulo, Brasil) ou Micromed Biotecnologia, modelo ErgoPC (Brasília, Brasil). Foram incluídos pacientes com idade igual e/ou superior a 40 anos, em ritmo sinusal, sem história clínica de AVE prévio ao primeiro ECG realizado. Traçados inválidos ou com problemas técnicos foram excluídos. Em relação aos pacientes que realizaram mais de um ECG no tempo de seguimento do estudo, os critérios para presença de CA foram analisados apenas no primeiro exame.

As informações clínicas, traçados de ECGs e relatórios foram armazenados em uma base de dados eletrônica da RTMG. Os laudos de ECG foram gerados em modelo de texto livre por cardiologistas e interpretados e codificados automaticamente em códigos de Glasgow e Minnesota pelo programa de análise de ECG de Glasgow 12 derivações (versão 28.4.1, emitido em 16 de junho de 2009).^17 , 18^

### Variáveis

Incluímos como variáveis de ajuste: dados clínicos e epidemiológicos, hipertrofia ventricular esquerda (HVE) ao ECG e doença arterial coronariana (DAC). O histórico clínico foi autorrelatado e coletado no momento do exame por meio de questionário padronizado. Dentre as variáveis clínicas incluídas estão: idade, sexo, hipertensão arterial, diabetes mellitus, dislipidemia, tabagismo e AVE prévio. HVE foi definida como hipertrofia ventricular com alterações de ST-T, em medidas eletrocardiográficas automatizadas obtidas pelo programa de análise de ECG de Glasgow de 12 derivações. DAC foi definida pela presença dos seguintes descritores na base de dados: “angioplastia coronariana”, “cardiopatia isquêmica crônica”, “cirurgia de revascularização miocárdica”, “infarto – angioplastia”, “infarto – clínico” e “síndrome coronariana aguda”.

CA foi definida pela presença das seguintes medidas da onda P: 1) Duração da onda P maior que 120 ms em todas as derivações e/ou; (2) PTFV1 maior que 4.000 µv × ms.

### Desfechos

O desfecho primário do estudo foi o desfecho composto de morte e/ou internação por AVE. Os desfechos secundários foram mortalidade geral, morte cardiovascular, FA incidente e síndrome coronariana aguda (SCA). Dados acerca dos desfechos foram obtidos por meio de pareamento probabilístico dos registros de ECG (contendo nome, sexo, data de nascimento e endereço) com dados de bancos de dados públicos: o Sistema de Informação de Mortalidade (SIM) e o Sistema de Informação Hospitalar (SIH). O pareamento foi realizado usando o software FRIL ( *fine-grained record linkage* ) software (version 2.1.5, Atlanta, GA).^19^

Os diagnósticos foram identificados usando códigos da Classificação Internacional de Doenças (CID-10) registrados no SIM e no SIH. Mortalidade por AVE foi definida pelos CID: ataques isquêmicos transitórios (G45; G45.9); hemorragia subaracnoide (I60.2; I60.4; I60.7; I60.9); hemorragia intracerebral (I61.3; I61.5; I61.8; I61.9); outras hemorragias intracranianas não traumáticas (I62.0; I62.9); infarto cerebral (I63; I63.4; I63.5; I63.8; I63.9); acidente vascular cerebral não especificado (I64); outras doenças cerebrovasculares (I67.1; I67.2; I67.8; I67.9); e sequelas de doenças cerebrovasculares (I69.0; I69.1; I69.2; I69.3; I69.4; I69.8). Para internações por AVE, consideramos os códigos do SIH: 303040149 e 303040300.

Para mortalidade por todas as causas, se considerou qualquer CID como causa básica do óbito. A mortalidade cardiovascular foi definida como óbito por um dos CID: I01-I01.9, I02.0, I05-I09.9; I20-I25.9; G45-G46.8, I60-I61.9, I62.0, I63-I63.9, I64, I65-I66.9, I67.0-I67.3, I67.5-I67.6, I68.1-I68.2, I69.0-I69.3, I11; A39.52, B33.2-B33.24, D86.85, I40-I43.9, I51.4-I51.5; I48, I71), I70.2-I70.7, I73-I73.9; e A39.51, I33-I33.9, I38-I39.9. A FA incidente foi definida como a presença de FA no laudo do cardiologista nos ECGs subsequentes e/ou a presença do CID I48 nos dados do SIH ou SIM. A SCA foi definida pela presença dos CIDs I20-I24 referentes à doença isquêmica aguda do coração, bem como registro dos códigos do SIH: 303060190 e 0303060280 e 406030049.

### Análise estatística

As características basais da população foram resumidas por estatística descritiva. Variáveis categóricas foram relatadas como contagens e porcentagens; variáveis contínuas foram apresentadas como médias e desvios padrão (DP). Comparações entre grupos foram feitas através do teste qui-quadrado para variáveis categóricas e do teste-T de Student não pareado para variáveis contínuas. A significância estatística foi definida como P<0.05 com intervalo de confiança (IC) de 95%. A normalidade da distribuição da amostra foi presumida devido ao tamanho amostral.

O *Hazard Ratio* foi calculado com o modelo de regressão multivariada de Cox. Modelos de ajuste incremental foram utilizados para controlar variáveis de confusão, assim distribuídas: Modelo 1: não ajustado; Modelo 2: ajustado para sexo e idade; Modelo 3: modelo 2 + ajuste para hipertensão arterial, diabetes mellitus, tabagismo, dislipidemia; Modelo 4: modelo 3 + ajuste para HVE.

Em todos os modelos de regressão de Cox ajustados, a suposição de riscos proporcionais (RP) foi avaliada por meio do teste dos resíduos de Schoenfeld, que verifica a hipótese nula de que a razão de riscos de cada covariável permanece constante ao longo do período de seguimento.

O programa R (versão 3.4.3, Viena, Áustria) foi utilizado para análise estatística.

### Considerações éticas

Este estudo foi aprovado pelo Comitê de Ética da Universidade Federal de Minas Gerais sob o número de protocolo 68496317.7.00005149.

## Resultados

**Figura 1:**
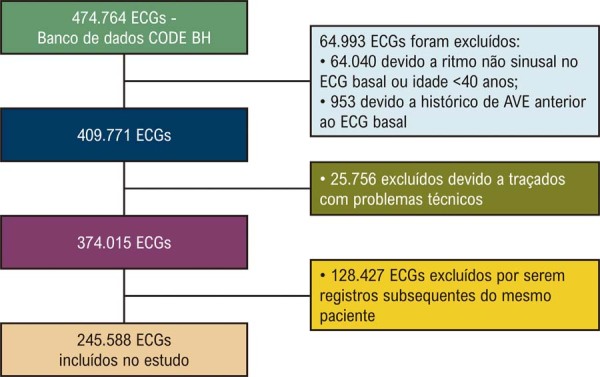
– Fluxograma ilustrando a seleção de pacientes de acordo com os critérios de exclusão. Fonte: Figura elaborada pelos autores.

Um total de 474.764 ECGs foi obtido na base CODE-BH. Após aplicação dos critérios de exclusão, 245.588 foram incluídos no estudo, conforme descrito na ( Figura 1 ).

As características basais dos pacientes incluídos no estudo estão expostas na Tabela 1 . Sexo masculino, hipertensão arterial, diabetes mellitus, dislipidemia e HVE estiveram associados de maneira significativa ao aumento em ambos os parâmetros da onda P, enquanto o tabagismo esteve relacionado apenas ao aumento na duração da onda P, e DAC não esteve associada de maneira significativa às alterações da onda P.

**Tabela 1:** – Características basais da população estudada, de acordo com a presença de cardiomiopatia atrial

	Total	Duração da Onda P		Valor p	PTFV1		Valor p
	(n=245588)	≤ 120 ms (n=180946)	> 120 ms (n=64642)		≤ 4.000 µv × ms (n=220846)	> 4.000 µv × ms (n=24742)	
Idade (anos), média (DP)	59,5 (11,7)	58,9 (11,7)	61,2 (11,8)	<0.001	59,6 (11,7)	59,4 (11,7)	0,07
Sexo masculino, n (%)	89639 (36.5)	61662 (34,1)	27.867 (43,1)	<0.001	79460 (36)	10069 (40,7)	<0,001
Hipertensão, n (%)	93418 (38)	66018 (36,5)	27.400 (43,1)	<0.001	82816 (37,5)	10602 (42,9)	<0,001
Diabetes mellitus, n (%)	39702 (16,2)	28363 (15,7)	11.339 (17,5)	<0.001	35253 (16)	4449 (18,0)	<0,001
Tabagismo, n (%)	19584 (8)	15144 (8,4)	4440 (6,9)	<0.001	17583 (8)	2001 (8,1)	0,49
Dislipidemia, n (%)	23214 (9,5)	16535 (9,1)	6.679 (10,3)	<0.001	20639 (9,3)	2575 (10,4)	<0,001
Hipertrofia Ventricular Esquerda, n (%)	4172 (17)	2687 (1,5)	1485 (2,3)	<0.001	3508 (1,6)	664 (2,7)	<0,001
Doença arterial coronariana, n (%)	1016 (0,4)	711 (0,4)	305 (0,5)	0.007	899 (0,4)	117 (0,5)	0,13

DP: desvio padrão; PTFV1: Força terminal da onda P na derivação V1 (P-wave terminal force in V1 lead).

Fonte: Tabela elaborada pelos autores.

O tempo de seguimento médio dos pacientes na base final foi de 3,5 anos com desvio padrão de 2,3. O desfecho de mortalidade ocorreu em 10.980 (4,5%) pacientes, internação em 80.718 (32,9%) pacientes, mortalidade cardiovascular em 1.753 (0,7%) pacientes, SCA em 1.016 (0,4%) pacientes e FA incidente em 316 (0,1%) pacientes. O desfecho primário de internação ou morte por AVE ocorreu em 1.860 (0,8%) pacientes. A ocorrência dos desfechos primários e secundários na amostra está exposta na Tabela 2 . A duração da onda P > 120 ms e a PTFV1 > 4.000 µv × ms, analisadas como variáveis categóricas, estiveram associadas ao desfecho primário de morte ou internação por AVE em análise não ajustada, e a associação se manteve após ajuste para variáveis sociodemográficas, fatores de risco cardiovasculares e presença de HVE (HR 1,24; IC 95%: 1,12 – 1,36 e HR 1,20; IC 95%: 1,05 – 1,38, respectivamente).

**Tabela 2:** – Ocorrência dos desfechos primários e secundários nos subgrupos de marcadores de cardiomiopatia atrial

Desfecho	Duração da onda P		Valor p	PTFV1		Valor p
	< 120 ms (n=180.946)	> 120 ms (n=64.642)		≤ 4.000 µv × ms (n=220.846)	> 4.000 µv × ms (n=24.742)	
Internação ou morte por AVE, n (%)	1216 (0,7)	646 (1,0)	<0,001	1619 (0,7)	241 (1,0)	<0,001
Mortalidade geral, n (%)	7703 (4,3)	3287 (5,1)	<0,001	9684 (4,4)	1302 (5,3)	<0,001
Mortalidade cardiovascular, n (%)	1179 (0,7)	575 (0,9)	<0,001	1522 (0,7)	232 (0,7)	<0,001
Síndrome Coronariana Aguda, n (%)	711 (0,4)	305 (0,5)	0,007	899 (0,4)	117 (0,5)	0,13
Fibrilação Atrial Incidente, n (%)	199 (0,1)	117 (0,2)	<0,001	279 (0,1)	37 (0,1)	0,33

PTFV1: Força terminal da onda P na derivação V1 (P-wave terminal force in V1 lead); AVE: acidente vascular encefálico.

Fonte: Tabela elaborada pelos autores.

A avaliação da associação entre os achados eletrocardiográficos e os desfechos primários foi realizada por meio de um modelo de regressão multivariada, e a suposição de riscos proporcionais foi realizada com o teste de Schoenfeld, que indicou violação global significativa da suposição de RP no modelo da onda P para hospitalização ou óbito por AVE (p=0,0008) e mortalidade por todas as causas (p=0,0002). No modelo de AVE, essa violação foi atribuída à variação temporal dos efeitos da idade (p=0,0008) e da hipertensão arterial (p=0,0007). Para mortalidade por todas as causas, a não proporcionalidade foi explicada principalmente pela categoria de duração da onda P (p=0,0002) e pelo sexo (p=0,0032). No caso da mortalidade cardiovascular, SCA e FA incidente, o teste global apontou tendência marginal à violação (p = 0,064), associada de forma consistente à categoria de duração da onda P (p = 0,023) e à hipertensão arterial (p = 0,040).

No modelo de PTFV1, se observou um padrão altamente consistente de violação da suposição de riscos proporcionais entre os cinco desfechos cardiovasculares analisados. Embora o teste global para cada desfecho tenha evidenciado apenas tendência marginal à violação (p=0,064), duas covariáveis apresentaram violações significativas e recorrentes em todos os modelos: a categoria de duração da onda P (p=0,023) e a hipertensão arterial (p=0,040). Nenhuma outra covariável, incluindo idade, sexo ou demais comorbidades, demonstrou evidências significativas de não proporcionalidade na análise de PTFV1.

**Figura 2:**
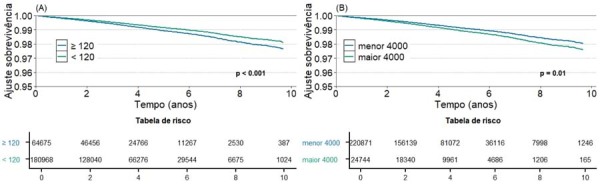
– Análise de sobrevivência para o desfecho primário de morte ou internação por AVE, em relação à exposição. a) Duração da onda P > 120ms; b) PTFV1 > 4.000 µv × ms. Fonte: Figura elaborada pelos autores.

**Figura 3:**
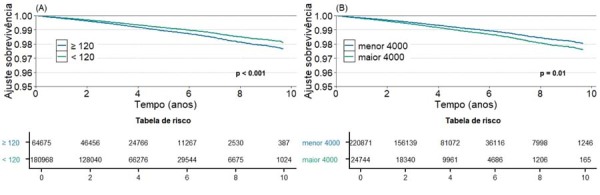
– Análise de sobrevivência para a exposição de duração da onda P > 120 ms para os desfechos secundários de: a) Morte Cardiovascular; b) FA incidente. Fonte: Figura elaborada pelos autores.

A análise de regressão multivariada realizada como descrito acima mostrou que o aumento na duração da onda P ajustada também esteve significativamente associado ao aumento dos desfechos de mortalidade cardiovascular e FA incidente, enquanto o aumento da PTFV1 ajustada também se associou a maior risco de morte e morte cardiovascular ( Tabelas 3 e 4 ). As curvas de análise de sobrevivência mostram uma dissociação dos eventos significativos nos pacientes estratificados pela presença ou ausência de CA, principalmente a partir do 4º ano de seguimento ( Figuras 2 , 3 e 4 ).

**Tabela 3:** – Associação entre o aumento da duração da onda P ao eletrocardiograma e os desfechos analisados

Desfecho	Modelo 1* HR (IC 95%)	Valor p (M1)	Modelo 2^**†**^ HR (IC 95%)	Valor p (M2)	Modelo 3^**‡**^ HR (IC 95%)	Valor p (M3)	Modelo 4^**§**^ HR (IC 95%)	Valor p (M4)
Internação ou morte por AVE	1,45 (1,32 – 1,6)	<0,001	1,25 (1,14 – 1,39)	<0,00	1,24 (1,13 – 1,37)	<0,001	1,24 (1,12 – 1,36)	<0,001
Mortalidade geral	1,17 (1,12 – 1,22)	<0,001	0,98 (0,94 – 1,02)	0,38	0,97 (0,95 – 1,03)	0,51	0,98 (0,94 – 1,02)	0,34
Mortalidade cardiovascular	1,33 (1,21 – 1,47)	<0,00	1,12 (1,01 – 1,24)	0,02	1,12 (1,02 – 1,24)	0,02	1,11 (1,01 – 1,23)	0,04
Síndrome Coronariana Aguda	1,2 (1,05 – 1,37)	0,008	1,06 (0,93 – 1,21)	0,4	1,04 (0,91 – 1,19)	0,56	1,04 (0,91 – 1,19)	0,62
Fibrilação Atrial incidente	1,6 (1,28 – 2,02)	<0,001	1,35 (1,08 – 1,70)	0,01	1,35 (1,08 – 1,70)	0,01	1,34 (1,06 – 1,68)	0,01

AVE: acidente vascular encefálico; HR: hazard ratio; IC: intervalo de confiança; *não ajustado; ^†^ajustado para sexo e idade; ^‡^ modelo 2 + ajustado para hipertensão, diabetes mellitus, tabagismo e dislipidemia; ^§^ modelo 3 + ajustado para hipertrofia ventricular esquerda.

Fonte: Tabela elaborada pelos autores.

**Tabela 4:** – Associação entre o aumento da PTFV1 ao eletrocardiograma e os desfechos analisados

Desfecho	Modelo 1* HR (IC 95%)	Valor p (M1)	Modelo 2^**†**^ HR (IC 95%)	Valor p (M2)	Modelo 3^**‡**^ HR (IC 95%)	Valor p (M3)	Modelo 4^**§**^ HR (IC 95%)	Valor p (M4)
Internação ou morte por AVE	1,24 (1,09 – 1,42)	0,01	1.23 (1,08 – 1,41)	0,002	1.22 (1,06 – 1,4)	0,004	1.2 (1,05 – 1,38)	0,01
Mortalidade geral	1,13 (1,07 – 1,2)	<0,001	1,12 (1,06 – 1,19)	<0,001	1,12 (1,06 –1,19)	<0,001	1,11 (1,05 – 1,17)	0,001
Mortalidade cardiovascular	1,28 (1,18 – 1,47)	<0,001	1,28 (1,11 – 1,49)	<0,001	1,27 (1,11 –1,46)	0,001	1,25 (1,09 – 1,43)	0,002
Síndrome Coronariana Aguda	1,16 (0,96 – 1,41)	0,13	1,12 (0,92 – 1,36)	0,25	1,08 (0,89 –1,31)	0,45	1,07 (0,88 – 1,29)	0,52
Fibrilação Atrial incidente	1,1 (0,78 – 1,55)	0,58	1,1 (0,78 – 1,55)	0,58	1,1 (0,78 –1,55)	0,58	1,08 (0,76 – 1,52)	0,67

AVE: acidente vascular encefálico; HR: hazard ratio; IC: intervalo de confiança; *não ajustado; ^†^ ajustado para sexo e idade; ^‡^ modelo 2 + ajustado para hipertensão, diabetes mellitus, tabagismo e dislipidemia; ^§^ modelo 3 + ajustado para hipertrofia ventricular esquerda.

Fonte: Tabela elaborada pelos autores.

**Figura 4:**
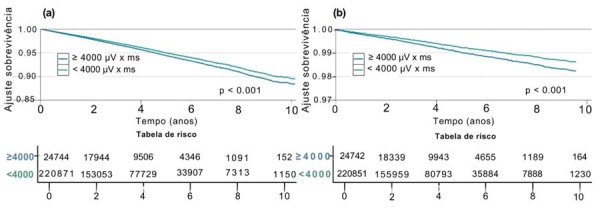
– Análise de sobrevivência para a exposição de PTFV1 > 4.000 µv × ms para os desfechos secundários de: a) Mortalidade Geral; b) Morte Cardiovascular. Fonte: Figura elaborada pelos autores.

## Discussão

Neste estudo observacional, encontramos associação significativa entre marcadores eletrocardiográficos de CA e um maior risco de internação ou morte por AVE. O aumento na duração da onda P ajustada também esteve associado a maior risco de morte cardiovascular e FA incidente, enquanto o aumento na PTFV1 ajustado esteve associado a aumento no risco de morte geral e morte cardiovascular. Comparado com estudos de coorte semelhantes, este estudo apresenta uma amostra significativamente maior e é o primeiro em uma população brasileira. O tempo de seguimento médio da base CODE-BH é menor (3,5 anos versus CHS: 12,9 anos; MESA: 8,5 anos; ARIC: 22 anos),^13 , 14 , 20^ o que poderia reduzir a chance de se encontrar os resultados esperados. Essa limitação pode ter sido contornada pela grande quantidade de pacientes incluídos no estudo.

Os nossos resultados corroboram evidências anteriores que associam CA a desfechos adversos.^14 , 21 , 22^ Os eventos isquêmicos cerebrovasculares demonstraram associação significativa com alterações eletrocardiográficas da onda P, as quais traduzem um substrato patológico no miocárdio atrial. Isso reforça a hipótese de que a CA pode estar relacionada a eventos tromboembólicos de maneira independente, não apenas mediada pela presença de FA ou outras cardiopatias descritas como de alto risco para o fenômeno trombogênico. Não obstante, as alterações da onda P também estiveram associadas a alguns dos desfechos não tromboembólicos do estudo, sugerindo que a CA pode ser um marcador de risco cardiovascular e de mortalidade. A CA ainda precisa ser melhor compreendida para que seu entendimento tenha impacto na prática clínica, como na estratificação de risco cardíaco.

A FA faz parte do espectro da CA e age como um mediador ou potencializador do fenômeno trombótico de maneira dose-dependente. Além de contribuir com a estase sanguínea, a CA parece estar relacionada à ativação e amplificação do processo de disfunção endotelial que já existe no átrio doente, aumentando a inflamação, ativação plaquetária e da cascata de coagulação, o que favoreceria ainda mais um estado pró-trombótico.^23^ Ainda é necessário definir, porém, se a CA isolada sem FA seria capaz de desencadear a trombogênese de forma clinicamente relevante. As atuais evidências e nossos achados levam a crer que sim, o que desviaria o foco de busca nos eventos neurológicos isquêmicos para detecção não apenas de FA, mas de uma abordagem mais ampla da CA por meio de marcadores eletrocardiográficos e outros biomarcadores.^7 , 24^

Existem métodos mais avançados para detecção mais específica de alterações patológicas do miocárdio atrial. A ecocardiografia tridimensional com medida do *strain* atrial é capaz de detectar tanto a fibrose quanto a alteração da função sistólica atrial.^25^ A ressonância magnética cardíaca pode extrair informações relacionadas a morfologia do átrio, função atrial esquerda^20^ e detecção quantitativa de fibrose atrial.^26^ A tomografia computadorizada cardíaca faz análise tridimensional da morfologia do átrio e do apêndice atrial esquerdo,^26^ especificando o tipo morfológico do apêndice atrial esquerdo que pode ter maior risco para AVE isquêmico.^24^ Já foram demonstradas associações entre alterações detectadas por esses métodos e eventos cardiovasculares.^20 , 24 , 26 , 27^ No entanto, é importante ressaltar que a investigação se inicia com ECG e anormalidades eletrocardiográficas podem ser o gatilho para direcionar a propedêutica nos casos de AVE isquêmico.

O uso da inteligência artificial (IA) na detecção e predição de FA já foi descrito.^22 , 28 , 29^ É razoável especular que as alterações da onda P seriam possíveis marcadores de explicabilidade para predição de FA. A sua incorporação parece ser promissora para agregar a modelos de predição de risco de eventos tromboembólicos em pacientes com AVE criptogênico. Os métodos de avaliação de CA ainda precisam ser mais bem desenvolvidos para permitir a predição de risco trombótico com aplicabilidade clínica.

Os achados deste estudo corroboram evidências disponíveis e permitem a geração de novas hipóteses relativas à associação entre CA e desfechos adversos. Entretanto, seus resultados não permitem a elaboração de novas estratégias de anticoagulação, em função de suas limitações. Trata-se de coorte retrospectiva com dados clínicos autorreferidos, o que introduz potencial viés de confusão, pois exposições específicas podem não ter sido incluídas no questionário. A amostra foi extraída da atenção primária e pacientes que não estavam em ritmo sinusal foram excluídos, o que pode limitar a generalização dos achados e restringir sua extrapolação para populações com risco cardiovascular presumidamente mais elevado. A incidência de FA pode ter sido subdimensionada, uma vez que a avaliação de ECGs únicos pode ter impedido a detecção de FA paroxística.

Os desfechos foram coletados de sistemas de informações do SUS de Belo Horizonte, que apresentam problemas de preenchimento dos dados e que se limitam apenas ao sistema público para as hospitalizações. Eventos ocorridos fora de Belo Horizonte, como internações ou óbitos, não foram computados. Esse aspecto do estudo também limitou a distinção entre os AVEs de mecanismo aterotrombótico de cardioembólico, uma vez que a CA parece ter papel central nos AVEs cardioembólicos. Essa diferenciação não foi possível devido à ausência de dados sobre o mecanismo subjacente nos bancos utilizados, bem como à inviabilidade de ajustamento para aterosclerose extracoronária, o que pode introduzir o viés de classificação.

O método de pareamento dos dados por alta probabilidade (acima de 95%) também traz limitações, devido ao risco de perda de pares por erros ou diferenças de cadastro e de pareamento incorreto por indivíduos homônimos com a mesma data de nascimento. O software da Universidade de Glasgow foi utilizado para todas as análises eletrocardiográficas. Embora seja uma ferramenta automática, podendo apresentar imprecisões em relação às medições manuais de cardiologistas, sua facilidade e disponibilidade de reprodutibilidade em diferentes cenários clínicos favorecem a incorporação na prática clínica.

## Conclusão

O aumento na duração da onda P > 120 ms e da PTFV1 > 4.000 µV × ms ajustado para fatores demográficos e fatores de risco cardiovasculares (HAS, DM, tabagismo, dislipidemia, DAC e HVE) esteve associado à maior incidência de morte ou internação por AVE. A duração aumentada da onda P teve associação ajustada também à morte cardiovascular e à FA incidente, e a PTFV1 aumentada também foi associada de forma ajustada à mortalidade geral e à morte cardiovascular. Este estudo compreende o maior número de pacientes já realizado, o que corrobora a hipótese de relação independente entre a CA e desfechos adversos. A partir de melhor compreensão dessa entidade e da identificação de novos biomarcadores, seria possível predizer eventos tromboembólicos com maior acurácia, o que permitiria o desenvolvimento de novas estratégias de anticoagulação direcionadas a indivíduos sob alto risco desses eventos, reduzindo a carga da doença e a morbimortalidade na população.
