# Supplementary material for: Cardiomiopatia Atrial Detectada por Eletrocardiograma: Associação com Acidente Vascular Encefálico em uma Coorte Eletrônica Brasileira
Source: Arq Bras Cardiol. 2026 Apr 1;123(3):e20250625. [Article in Portuguese] doi: 10.36660/abc.20250625 (PMC13128205; doi:10.36660/abc.20250625)
Supplement: Translation

# Translation: Atrial Cardiomyopathy Detected by Electrocardiogram: Association with Stroke in a Brazilian Electronic Cohort

**DOI:** 10.36660/abc.20250625i

**Authors:** Filipe Antunes de Lêu, Gabriela Miana de Mattos Paixão, Ana Cecilia Oliveira, Carla Paula Moreira Soares, Paulo R. Gomes, Peter Macfarlane, Antônio Luiz Pinho Ribeiro

**Affiliations:** 1 Brazil Universidade Federal de Minas Gerais, Belo Horizonte, MG – Brazil; 2 England University of Glasgow School of Health and Wellbeing, Glasgow, – England

**Keywords:** Cardiomyopathies, Electrocardiography, Stroke, Mortality, Cohort Studies

## Abstract

**Background:**

Atrial cardiomyopathy (AC) is a condition detectable by electrocardiogram (ECG) that may play a significant role in the pathophysiology of the etiology of strokes, independently of atrial fibrillation (AF).

**Objectives:**

To evaluate the association between ECG markers of AC (prolonged P-wave duration >120 ms and P-terminal force in V1 (PTFV1) >4,000 µV·ms) as well as the occurrence of mortality and hospitalization due to stroke.

**Methods:**

This retrospective cohort study included patients from Belo Horizonte who underwent ECGs between 2006 and 2018. Patients aged ≥40 years, in sinus rhythm, and without a previous history of stroke at baseline ECG were included. Clinical and ECG data were linked to mortality (SIM) and hospitalization (SIH) public databases. Cox regression was used to calculate hazard ratios (HRs), and incremental adjustment models for age, sex, cardiovascular risk factors, and left ventricular hypertrophy were employed. Statistical significance was set at p<0.05.

**Results:**

245,588 patients were included. 26.3% had prolonged P-wave duration >120 ms, and 10.1% had elevated PTFV1 >4,000 µV·ms. Mean follow-up duration was 3.5 years. AC was associated with mortality and hospitalization due to stroke (HR 1.24; 95% CI, 1.12–1.36 for P-wave duration >120 ms; p<0.001; HR 1.20; 95% CI, 1.05–1.38 for PTFV1 >4,000 µV·ms; p<0.001).

**Conclusion:**

ECG markers of AC are associated with death or hospitalization due to stroke, as well as cardiovascular mortality and incident AF in a large, representative Brazilian cohort, highlighting their prognostic value.

## Introduction

Stroke is the third leading cause of death globally, with a significant socioeconomic impact.^1 , 2^Ischemic stroke accounts for 87% of these cases, and its etiology is linked to atherothrombotic and cardioembolic events.^3^Despite adequate investigation, the etiology is not properly clarified in 25% of these cases, which are referred to as cryptogenic strokes. In such cases, a knowledge gap exists regarding optimal secondary prevention strategies, resulting in an average recurrence rate of 3-6% of new events per year.^4 , 5^Emerging evidence suggests that a portion of cryptogenic stroke cases are caused by cardioembolic or atheroembolic mechanisms.^6^In this scenario, atrial cardiomyopathy (AC) may play a causal role in cardioembolic strokes.^7^

AC is characterized by structural and functional endocardial changes driven by several genetic and environmental stimuli, including age and cardiovascular risk factors.^8 , 9^Traditionally, it is considered a marker or a consequence of other cardiac conditions, as a precursor of atrial fibrillation (AF). However, the paradigm of this chain of events has been evolving in light of evidence that AC may be related to ischemic events, in spite of the presence of AF.^7^AC disrupts homeostasis, which triggers fibroproliferative signalling pathways and leads to various cardiomyocyte alterations, resulting in endothelial dysfunction, inflammation, hypocontractility, and the release of procoagulant factors, ultimately contributing to thrombogenesis.^8^While AF enhances this cascade, as it accelerates atrial remodelling and increases blood stasis, its presence is not essential for the occurrence of thromboembolic events.

The 12-lead electrocardiogram (ECG) is a simple, non-invasive, and widely available diagnostic tool that may be useful in the detection of AC through an increase in P-wave duration and an increase of P-wave terminal force in lead V1 (PTFV1) - a product of duration and amplitude of the terminal P-wave negative phase in V1.^10 , 11^Observational studies in structured databases suggest a link between AC in ECG and adverse outcomes, such as ischemic stroke.^12 - 14^In this context, we aimed to assess the association between cardiovascular events and death in an electronic Brazilian cohort ( Central Illustration ).

## Methods

### Study Design

This retrospective cohort study comprises the analysis of a subgroup from the Clinical Outcomes in Digital Electrocardiography (CODE) cohort, previously described.^15^The findings were reported in accordance with the STROBE (Strengthening the Reporting of Observational Studies in Epidemiology) guidelines.^16^

For the present analysis, patients who had undergone ECGs between 2006 and 2018 in the city of Belo Horizonte, which is part of the Rede de Telessaúde de Minas Gerais (RTMG),^17^were included in the study. This subgroup of ECGs performed in Belo Horizonte was named CODE-BH.

ECGs were performed in primary care settings as deemed necessary by the patient’s attending clinician using digital devices (Tecnologia Eletrônica Brasileira, ECGPC, São Paulo, Brazil, or Micromed Biotecnologia, ErgoPC, Brasília, Brazil). Patients aged ≥40 years old, in sinus rhythm, and without a history of stroke before ECG recording were considered eligible. ECGs with artifacts or changes that precluded analysis were excluded. For patients who underwent more than one ECG recording during follow-up, only the first was included.

Recorded ECGs and patients’ records were stored in an electronic database of the RTMG. Subsequently, they were appraised by cardiologists, who generated reports in free-text models, and also underwent interpretation by the University of Glasgow’s automated ECG analysis program, which included automated coding based on the Minnesota Code.^17 , 18^

### Variables

For adjustment variables, we included clinical and epidemiological data, left ventricular hypertrophy (LVH), and coronary artery disease (CAD). Epidemiological and clinical data were obtained through a self-reported, standardized questionnaire performed at the time of electrocardiography, encompassing age, sex, hypertension, dyslipidemia, smoking history, and previous stroke. LVH was defined as ventricular hypertrophy with ST-T changes in electrocardiographic measurements obtained by the Glasgow program. CAD was defined by the presence of any of the following database descriptors: “coronary angioplasty”, “chronic ischemic heart disease”, “coronary artery bypass grafting”, “heart attack – angioplasty”, “heart attack – clinical”, and “acute coronary syndrome”.

AC was defined by the presence of the following P-wave measures: 1) P-wave duration > 120 ms based on all leads and/or 2) P-wave terminal force in V1 (PTFV1) > 4.000 µv × ms.

### Outcomes

The primary outcome was a composite endpoint of death and/or admission on account of stroke. Secondary outcomes included all-cause mortality, cardiovascular death, AF, and acute coronary syndrome (ACS). Outcome data were obtained through a probability pairing of ECG records (containing name, sex, date of birth, and address) with data from databases of the Brazilian Unified Health System (SUS): the System of Information on Mortality (SIM) and the Hospital Information System (SIH). Probability pairing was performed using FRIL (fine-grained record linkage) software (version 2.1.5, Atlanta, GA).^19^

Diagnoses were identified using the International Classification of Diseases (ICD-10), coded in SIM and SIH databases. Mortality caused by stroke was defined by ICD-10 codes: Transient Ischemic Attacks (G45; G45.9); Subarachnoid Hemorrhage (I60.2; I60.4; I60.7; I60.9); Intracerebral Hemorrhage (I61.3; I61.5; I61.8; I61.9); Other Nontraumatic Intracranial Hemorrhage (I62.0; I62.9); Cerebral Infarction (I63; I63.4; I63.5; I63.8; I63.9); Not specified Stroke (I64); Other Cerebrovascular diseases (I67.1; I67.2; I67.8; I67.9); Sequelae of Cerebrovascular Disease (I69.0; I69.1; I69.2; I69.3, I69.4 and I69.8). Stroke admissions were identified using the Brazilian hospitalization codes 303040149 and 303040300.

All-cause mortality was defined as death caused by any ICD code. Cardiovascular death was determined by the presence of any of the following ICD codes: rheumatic heart disease (I01-I01.9, I02.0, I05-I09.9), ischemic heart disease (I20-I25.9), cerebrovascular disease (G45-G46.8, I60-I61.9, I62.0, I63-I63.9, I65-I66.9, I67.0-I67.3, I67.5-I67.6, I68.1-I68.2, I69.0-I69.3), hypertensive heart disease (I11), myocarditis (A39.52, B33.2-B33.24, D86.85, I40-I43.9, I51.4-I51.5), AF or flutter (I48), aortic aneurysm (I71), peripheral artery disease (I70.2-I70.7, I73-I73.9) and endocarditis (A39.51, I33-I33.9, I38-I39.9). Incident AF was determined by its documentation in a cardiologist’s report on subsequent ECGs and/or the presence of the ICD-10 code I48 in SIH or SIM data. ACS was identified by ICD-10 codes I20-I24 for acute ischemic heart disease and SIH codes for acute myocardial infarction and/or ACS treatment (303060190 and 0303060280) and primary percutaneous coronary intervention (0406030049).

### Statistical analysis

Descriptive statistics were used to summarize the characteristics of the study population. Categorical variables were reported in frequencies and percentages, while continuous variables were presented as means and standard deviations (SD). Comparisons between groups were made with the chi-square test for categorical variables and with an unpaired Student’s T test for continuous variables. Statistical significance was set at p<0.05 with a 95% confidence interval (CI). Normal distribution of the sample was assumed due to the sample size.

Hazard Ratio (HR) was calculated with a Cox multivariate regression model. Incremental adjustment models were employed to control for confounding variables, distributed as follows: Model 1: unadjusted; Model 2: adjusted for sex and age; Model 3: Model 2 + adjustment for arterial hypertension, diabetes mellitus, smoking, and dyslipidemia; Model 4: Model 3 + LVH. The adequacy of the regression model was assessed using the Schoenfeld residuals test. For all adjusted Cox regression models, the proportional hazards (PH) assumption was evaluated using the Schoenfeld residuals test, which formally tests the null hypothesis that the hazard ratio for each covariate remains constant throughout the follow-up period.

All analyses were conducted using the R software (version 3.4.3, Vienna, Austria).

### Ethical considerations

This study was approved by the Research Ethics Committee of the Federal University of Minas Gerais (COEP-UFMG), protocol number 68496317.7.00005149.

## Results

The CODE-BH database comprised 474,764 ECGs. After excluding those that met exclusion criteria and those that were second or subsequent ECGs from the same patient, the final sample included 245,588 patients ( Figure 1 ).

**Figure 1:** – Flowchart illustrating patient selection according to exclusion criteria. Source: Figure elaborated by the authors.

The baseline characteristics of the study participants are presented in Table 1 . Male sex, hypertension, diabetes mellitus, dyslipidemia, and LVH were significantly associated with an increase in both P-wave parameters, whereas smoking was associated only with prolonged P-wave duration, and CAD was not significantly associated with P-wave abnormalities.

**Table 1:** – Baseline Characteristics of the Study Population According to the Presence of Atrial Cardiomyopathy

	Total	P-wave duration		p-value	PTFV1		p-value
	(n=245.588)	< 120 ms (n=180.946)	> 120 ms (n=64.642)		≤ 4.000 µv × ms (n=220.846)	> 4.000 µv × ms (n=24.742)	
Age (years), mean (SD)	59.54	58.9 (11.7)	61.2 (11.8)	<0.001	59.6 (11.7)	59.4 (11.7)	0.07
Male sex, n (%)	89 639 (36.5)	61 662 (34.1)	27 867 (43.1)	<0.001	79 460 (36)	10 069 (40.7)	<0.001
Hypertension, n (%)	93 418 (38)	66 018 (36.5)	27 400 (43.1)	<0.001	82 816 (37.5)	10 602 (42.9)	<0.001
Diabetes mellitus, n (%)	39 702 (16.2)	28 363 (15.7)	11 339 (17.5)	<0.001	35 253 (16)	4 449 (18.0)	<0.001
Smoking, n (%)	19 584 (8)	15 144 (8.4)	4 440 (6.9)	<0.001	17 583 (8)	2 001 (8.1)	0.49
Dyslipidemia, n (%)	23 214 (9.5)	16 535 (9.1)	6 679 (10.3)	<0.001	20 639 (9.3)	2 575 (10.4)	<0.001
Left Ventricle Hypertrophy, n (%)	4 172 (17)	2 687 (1.5)	1 485 (2.3)	<0.001	3 508 (1.6)	664 (2.7)	<0.001
Arterial coronary disease, n (%)	1 016 (0.4)	711 (0.4)	305 (0.5)	7,000	899 (0.4)	117 (0.5)	0.13

SD: standard deviation; PTFV1: P-wave terminal force in V1 lead.

Source: Table elaborated by the authors.

The mean follow-up duration for patients in the final cohort was 3.5 years (standard deviation, 2.3). Mortality occurred in 10,980 patients (4.5%), hospitalization in 80,718 patients (32.9%), cardiovascular mortality in 1,753 patients (0.7%), acute coronary syndrome (ACS) in 1,016 patients (0.4%), and incident AF in 316 patients (0.1%). The primary outcome of hospitalization or death due to stroke occurred in 1,860 patients (0.8%). The occurrence rate of primary and secondary outcomes is presented in Table 2 . P-wave duration >120 ms and P-terminal force in V1 (PTFV1) >4,000 µV·ms, analyzed as categorical variables, were associated with the primary outcome of death or hospitalization due to stroke in unadjusted analyses, with the association persisting after incremental adjustments for sociodemographic variables, cardiovascular risk factors, and left ventricular hypertrophy (HR 1.24; 95% CI, 1.12–1.36 and HR 1.20; 95% CI, 1.05–1.38, respectively).

**Table 2:** – Occurrence of primary and secondary outcomes in subgroups of Atrial Cardiomyopathy markers

	P-wave duration		p-value	PTFV1		p-value
Outcome	< 120 ms (n=180.946)	> 120 ms (n=64.642)		≤ 4.000 µv × ms (n=220.846)	> 4.000 µv × ms (n=24.742)	
Hospitalization or death due to stroke, n (%)	1216 (0.7)	646 (1.0)	<0.001	1619 (0.7)	241 (1.0)	<0.001
All-cause mortality, n (%)	7703 (4.3)	3287 (5.1)	<0.001	9684 (4.4)	1302 (5.3)	<0.001
Cardiovascular mortality, n (%)	1179 (0.7)	575 (0.9)	<0.001	1522 (0.7)	232 (0.7)	<0.001
Acute Coronary Syndrome, n (%)	711 (0.4)	305 (0.5)	0.007	899 (0.4)	117 (0.5)	0.13
Incident Atrial Fibrillation, n (%)	199 (0.1)	117 (0.2)	<0.001	279 (0.1)	37 (0.1)	0.33

PTFV1: P-wave terminal force in V1 lead.

Source: Table elaborated by the authors.

Analysis of the association between ECG findings and primary and secondary outcomes was performed with a Cox multivariate regression model, and the Proportional Hazards assumption was assessed with the Schoenfeld residuals test, which indicated a significant global violation of the Proportional Hazards (PH) assumption in the P-wave model for hospitalization or death due to stroke (p=0.0008) and all-cause Mortality (p=0.0002). In the stroke model, this violation was attributed to time-varying effects of age (p=0.0008) and arterial hypertension (p=0.0007). For all-cause mortality, non-proportionality was driven by the P-duration category (p=0.0002) and sex (p=0.0032). For cardiovascular mortality, acute coronary syndrome (ACS), and incident AF, the global test showed a marginal trend toward violation (p=0.064), consistently associated with PH violations related to the P-duration category (p=0.023) and arterial hypertension (p=0.040). In the PTFV1 model, a highly consistent pattern of PH assumption violation was observed across all five cardiovascular endpoints. Although the global test for each endpoint showed only a marginal trend toward violation (p = 0.064), two covariates consistently exhibited significant individual violations across all models: P-wave duration category (p = 0.023) and arterial hypertension (p = 0.040). No other covariates, including age, sex, or other comorbidities, showed significant evidence of non-proportionality in the PTFV1 analysis.

Multivariate regression analysis performed as described showed that adjusted prolonged P-wave duration was significantly associated with increased risks of cardiovascular mortality and incident AF, while adjusted elevated PTFV1 was also associated with higher risk of all-cause mortality and cardiovascular mortality ( Tables 3 and 4 ).

**Table 3:** – Relationship between P-wave Duration>120 ms on Electrocardiogram and outcomes

Outcome	Model 1* HR (CI 95%)	p value (M1)	Model 2^**†**^HR (CI 95%)	p value (M2)	Model 3^**‡**^HR (CI 95%)	p-value (M3)	Model 4^**§**^HR (CI 95%)	p-value (M4)
Hospitalization or death due to stroke	1.45 (1.32 – 1.6)	<0.001	1.25 (1.14 – 1.39)	<0.001	1.24 (1.13 – 1.37)	<0.001	1.24 (1.12 – 1.36)	<0.001
All-cause mortality	1.17 (1.12 – 1.22)	<0.001	0.98 (0.94 – 1.02)	0.38	0.97 (0.95 – 1.03)	0.51	0.98 (0.94 – 1.02)	0.34
Cardiovascular mortality	1.33 (1.21 – 1.47)	<0.001	1.12 (1.01 – 1.24)	0.02	1.12 (1.02 – 1.24)	0.02	1.11 ( 1.01 – 1.23)	0.04
Acute Coronary Syndrome	1.2 (1.05 – 1.37)	0.008	1.06 (0.93 – 1.21)	0.4	1.04 (0.91 – 1.19)	0.56	1.04 (0.91 – 1.19)	0.62
Incident Atrial Fibrillation	1.6 (1.28 – 2.02)	<0.001	1.35 (1.08 – 1.70)	0.01	1.35 (1.08 – 1.70)	0.01	1.34 (1.06 – 1.68)	0.01

HR: hazard ratio; CI: confidence interval; *unadjusted;^†^adjusted for sex and age;^‡^model 2 + adjusted for hypertension, diabetes mellitus, smoking, and dyslipidemia;^§^model 3 + adjusted for left ventricular hypertrophy.

Source: Table elaborated by the authors.

**Table 4:** – Relationship between Elevated P-terminal Force in V1 (PTFV1) on Electrocardiogram and Outcomes

Outcome	Model 1* HR (CI 95%)	p-value (M1)	Model 2^**†**^HR (CI 95%)	p-value (M2)	Model 3^**‡**^HR (CI 95%)	p-value (M3)	Model 4^**§**^HR (CI 95%)	p-value (M4)
Hospitalization or death due to stroke	1.24 (1.09 – 1.42)	0.01	1.23 (1.08 – 1.41)	0.002	1.22 (1.06 – 1.4)	0.004	1.2 (1.05 – 1.38)	0.01
All-cause mortality	1.13 (1.07 – 1.2)	<0.001	1.12 (1.06 – 1.19)	<0.001	1.12 (1.06 –1.19)	<0.001	1.11 (1.05 – 1.17)	0.001
Cardiovascular mortality	1.28 (1.18 – 1.47)	<0.001	1.28 (1.11 – 1.49)	<0.001	1.27 (1.11 –1.46)	0.001	1.25 (1.09 – 1.43)	0.002
Acute Coronary Syndrome	1.16 (0.96 – 1.41)	0,13	1.12 (0.92 – 1.36)	0.25	1.08 (0.89 –1.31)	0.45	1.07 (0.88 – 1.29)	0.52
Incident Atrial Fibrillation	1.1 (0.78 – 1.55)	0.58	1.1 (0.78 – 1.55)	0.58	1.1 (0.78 –1.55)	0.58	1.08 (0.76 – 1.52)	0.67

HR: hazard ratio; CI: confidence interval; *unadjusted;^†^adjusted for sex and age;^‡^model 2 + adjusted for hypertension, diabetes mellitus, smoking, and dyslipidemia;^§^model 3 + adjusted for left ventricular hypertrophy.

Source: Table elaborated by the authors.

Survival analysis curves demonstrated a significant divergence in event rates among patients stratified by the presence or absence of AC, with a more pronounced divergence observed from the fourth year of follow-up onward ( Figures 2 , 3 , and 4 ).

**Figure 2:** – Survival analysis for the primary outcome of death or hospitalization due to stroke in relation to exposure. (a) P-wave duration >120 ms; (b) P-terminal force in V1 (PTFV1) >4,000 µV·ms.

**Figure 3:** – Survival analysis for P-wave duration >120 ms in relation to secondary outcomes. (a) Cardiovascular mortality; (b) Incident atrial fibrillation.

**Figure 4:** – Survival analysis for P-terminal force in V1 (PTFV1) >4,000 µV·ms in relation to secondary outcomes. (a) All-cause mortality; (b) Cardiovascular mortality.

## Discussion

In this cohort study, we identified a significant, independent association between electrocardiographic markers of AC (increase in P-wave duration and PTFV1) and an increased risk of stroke-related death and hospitalization. P-wave duration>120 ms was also associated with cardiovascular mortality and incident AF, while elevated PTFV1 was linked to all-cause mortality and cardiovascular mortality. When compared to cohort studies with similar designs, the present research features the largest sample and is the first to be conducted in a Brazilian population. We expected that a shorter mean follow-up time when compared with other cohorts (3.5 years vs CHS: 12,9 years; MESA: 8,5 years; ARIC: 22 years)^13 , 14 , 20^would reduce the chance of events. This limitation, however, has been overcome by the larger sample size.

Our results corroborate prior evidence linking AC to adverse outcomes.^13 , 21 , 22^In this context, ischemic cerebrovascular events stand out, having shown a significant association with P-wave alterations, which translates to underlying atrial myocardial pathology. These findings validate the hypothesis that AC may be associated with thromboembolic events through a direct, independent pathway, not only mediated by the presence of AF or other cardiomyopathies described as a risk factor for thrombogenesis. Furthermore, P-wave alterations were also associated with outcomes other than thromboembolic, hence suggesting that AC may be a marker of cardiovascular risk and mortality. AC remains incompletely understood, highlighting the need for further research due to its potential to impact clinical practice as a predictor of cardiovascular risk.

AF is a part of the AC spectrum and plays a role as a mediator or enhancer of thromboembolic events in a dose-dependent fashion. Besides contributing to blood stasis, AC may be related to activation and amplification of already existing atrial endothelial dysfunction, hence increasing inflammation, platelet activation, and the coagulation cascade, favoring prothrombotic states.^23^The question of whether AC without AF is a sufficient cause of clinically relevant thrombogenesis remains unsolved. However, available evidence and our findings suggest this possibility, which may pave the way for a shift in the approach to preventing and investigating ischemic neurological events, and towards broader AC detection strategies via ECG and other biomarkers, beyond AF alone.^7 , 24^

Several methods have been studied to improve the detection of AC. Tridimensional echocardiography with atrial strain measurements is an evolving technique, able to detect both atrial fibrosis and systolic dysfunction through analysis of segmental longitudinal shortening and stretching of the atrial myocardium.^25^Magnetic cardiac resonance may provide information on atrial morphology, left atrial (LA) function through phasic variations in volume, contraction, and segmental shortening, and LA emptying fraction,^20^as well as quantitative detection of atrial fibrosis through late-enhancement with gadolinium.^26^Computerized cardiac tomography can perform tridimensional analysis of atrial morphology and LA appendix,^26^and specific LA morphologic types have been associated with increased risk of ischemic stroke.^24^The use of such methods has shown association with relevant outcomes, e.g., AF, mortality, cardiovascular events, and ischemic stroke.^20 , 24 , 26 , 27^However, ECG remains the initial, widely available tool for cardiomyopathy investigation, and the recognition of its electrocardiographic patterns should trigger an extensive diagnostic approach, mainly in cryptogenic ischemic stroke investigation.

The use of artificial intelligence (AI) in the detection and prediction of AF has already been described.^22 , 28 , 29^It is reasonable to speculate that P-wave alterations may explain the results obtained with these models for the prediction of AF. The use of AI in ECG interpretation seems to be a promising tool to improve prediction models of thromboembolic events in patients with cryptogenic stroke. AC assessment methods still require further exploration in order to allow thrombotic risk prediction and clinical applicability.

This study, to the best of our knowledge, is the first to assess AC using the ECG in a large cohort of Brazilian patients. Our findings align with and corroborate existing evidence and enable the generation of new hypotheses regarding the association between AC and adverse outcomes, as well as the development of new risk prediction tools. However, our results are not yet sufficient to support new anticoagulation strategies, as they are subject to several limitations that must be addressed. First, this work is a retrospective cohort study relying on self-reported clinical data, which introduces the possibility of confounding bias. Specific exposures and risk factors may not have been captured in the questionnaire, which could affect the comprehensiveness of the data. Besides, our sample was drawn from a primary care setting, and patients who were not in sinus rhythm were excluded, which may limit the generalizability of our findings and constrain their extrapolation to populations with a presumably higher cardiovascular risk. Incident AF may also have been underestimated due to reliance on single ECG assessments, potentially hindering the detection of paroxysmal AF.

Outcome data were obtained from public health system information systems in Belo Horizonte. This aspect of the study limits our ability to separate strokes caused by atherothrombotic from cardioembolic mechanisms, since AC may play a significant role in cardioembolic strokes. This differentiation was hindered by the absence of information on the underlying mechanisms of strokes in the database from which this data was extracted, as well as the inability to adjust for extracoronary atherosclerosis, which may introduce misclassification bias. Also, this data is subject to errors in documentation, while records of hospital admissions are limited to public hospitals. Furthermore, outcomes that occurred outside Belo Horizonte, such as hospital admissions and deaths, were not included in these records, potentially underestimating the total number of events. Additionally, our data identification process relies on high-probability linkage (over 95% of matches) that links patients across databases by name, sex, and age. However, this strategy introduces the risk of pairs’ loss due to misspellings or incorrect pairings based on homonyms among patients with the same birth date. Furthermore, the violation of the proportional hazards assumption for a few covariates, as described in the Methods section, may limit the interpretability of the hazard ratios reported in our analysis. Finally, the University of Glasgow software was used for all electrocardiographic analyses. Although it is an automated tool with the potential for inaccuracies compared to manual measurements by cardiologists, its ease of use and reproducibility across various clinical settings facilitate its incorporation into clinical practice.

## Conclusion

Electrocardiographic markers of AC (P-wave duration >120 ms and PTFV1>4000 µV × ms) adjusted to demographic data and cardiovascular risk factors are associated with increased risk of stroke-related death and hospitalization. Prolonged P-wave duration is also linked to cardiovascular mortality and incident AF, while elevated PTFV1 was also associated with all-cause mortality and cardiovascular mortality. To our knowledge, this study included the largest sample size among studies with a similar design performed to date, which strengthens the hypothesis of an independent association between AC and adverse clinical outcomes. Further research is needed to clarify the understanding of AC as a pathological entity and to identify biomarkers able to predict thromboembolic events. Such achievements could enhance risk stratification tools and anticoagulation strategies for high-risk individuals, hence reducing mortality and the burden of disease.
